# Genetic Burden Contributing to Extremely Low or High Bone Mineral Density in a Senior Male Population From the Osteoporotic Fractures in Men Study (MrOS)

**DOI:** 10.1002/jbm4.10335

**Published:** 2020-01-22

**Authors:** Shan Chen, Mahim Jain, Shalini Jhangiani, Zeynep C Akdemir, Philippe M Campeau, Robert F Klein, Carrie Nielson, Hongzheng Dai, Donna M Muzny, Eric Boerwinkle, Richard A Gibbs, Eric S Orwoll, James R Lupski, Jennifer E Posey, Brendan Lee

**Affiliations:** ^1^ Department of Molecular and Human Genetics Baylor College of Medicine Houston TX USA; ^2^ Osteogenesis Imperfecta Clinic, Kennedy Krieger Institute Baltimore MD USA; ^3^ Human Genome Sequencing Center Baylor College of Medicine Houston TX USA; ^4^ University of Montreal Medical Genetics Service Montréal QC Canada; ^5^ School of Medicine Oregon Health & Science University Portland OR USA; ^6^ Human Genetics Center and Department of Epidemiology UTHealth School of Public Health Houston TX USA; ^7^ Department of Pediatrics Baylor College of Medicine and Texas Children's Hospital Houston TX USA

**Keywords:** DXA, ANALYSIS/QUANTITATION OF BONE, HUMAN ASSOCIATION STUDIES, GENETIC RESEARCH, OSTEOPOROSIS, DISEASES AND DISORDERS OF/RELATED TO BONE

## Abstract

Worldwide, one in five men aged over 50 years will experience osteoporosis or a clinical bone fracture, with a greater fracture‐related mortality rate than women. However, the genetic etiology of osteoporosis in men is still poorly understood. We aimed to identify the genetic variants and candidate genes associated with extremely low or high BMD for a better understanding of the biology underlying low bone density that may point to potential therapeutic targets for increasing bone mass. Subjects from the Osteoporotic Fractures in Men Study (MrOS) cohort were evaluated by age and BMI‐adjusted total hip BMD. Those with BMD values 3 SDs away from the mean were selected and the remaining individuals whose adjusted BMD ranked at the highest or lowest 100 were included. Men with the lowest adjusted BMD (*N* = 98) and highest adjusted BMD (*N* = 110) were chosen for exome sequencing. Controls (*N* = 82) were men of Northern and Western European descent from the US Utah population of the 1000 Genomes Project. Fisher's exact test was performed to compare low‐ or high‐BMD subjects with controls for single‐gene associations. Additionally, sets of candidate genes causative of heritable disorders of connective tissue, including osteogenesis imperfecta (OI) and Ehlers‐Danlos syndrome (EDS), were grouped for multigene and mutation burden analyses. No single‐gene associations with rare variants were found for either the low BMD group (33 genes) or high BMD group (18 genes). In the group of OI genes, we detected a significant threefold increased accumulation of rare variants in low‐BMD subjects compared with controls (*p* = 0.009). Additionally, genes associated with EDS had a twofold increased frequency in low‐BMD subjects compared with controls (*p* = 0.03). These findings reveal a rare variant burden in OI and EDS disease genes at low BMD, which suggests a potential gene‐panel approach to screen for multivariant associations in larger cohorts. © 2019 The Authors. *JBMR Plus* published by Wiley Periodicals, Inc. on behalf of American Society for Bone and Mineral Research.

## Introduction

Osteoporotic fractures (OFs) are defined as fractures specifically occurring at sites associated with low BMD.^1^ The lifetime risk for OFs is quite high and ranges between 40% and 50% in women and between 13% and 22% in men. The Osteoporotic Fractures in Men Study (MrOS) is a large prospective cohort study designed to understand the epidemiology, environmental, and genetic factors underlying OFs in men.^2^ One of the significant risk factors identified through this cohort is low BMD, which shows a consistent relationship with increased fracture risk that could impact multiple skeletal locations.^3^, ^4^, ^5^

BMD is a highly heritable trait, with an heritability estimate of 50% to 80%,^6^ depending on skeletal locations and ethnicity. Recently, a genome‐wide association study (GWAS) of BMD has been enabled by the release of large sources of public health records such as the UK Biobank and 23andMe summary data, resulting in a sample size between 100,000 and 500,000, nearly 10 times more than the average GWAS size. These large‐scale studies revealed over 500 loci for estimated BMD (eBMD) based on quantitative ultrasound of the heel, of which approximately 300 loci were novel. The known and novel loci in combination explained approximately 20% of the eBMD variance.^7^, ^8^ Dual‐energy X‐ray absorptiometry (DXA)‐derived BMD, used most often in a clinical setting, has been associated with 80 loci in the largest study to date, involving approximately 66,000 samples.^9^ It explained approximately 10% of total body BMD and identified 36 novel loci. GWASs on extremely high or low BMD have revealed additional loci such as *SPON1* and *NPR3*, demonstrating the merit of selection of a more homogeneous case group in terms of BMD distribution.^10^ These pivotal studies have fundamentally changed the landscape of the genetic discovery for bone mass.

Although we embrace the joy of comprehending genetic factors for BMD distribution at a population level, the search for causal mutations is still hampered by the fact that the studies relied on genotyping array and imputation for their design. Next‐generation sequencing technologies, including exome sequencing (ES) and whole‐genome sequencing (WGS), have emerged as powerful tools in the determination of genetic factors. Unlike SNP‐based GWASs, sequencing‐based GWASs can uncover low‐frequency or rare variants that have not been discovered by previous methods. A recent WGS‐based study on BMD identified two low‐frequency, noncoding variants with an effect size fourfold greater than ever reported for common variants for lumbar spine BMD.^11^ One of the variants is a low‐frequency SNP (rs11692564) near the *EN1* gene. *En1* ^*−/−*^ mice recapitulated the low‐bone‐mass phenotype observed in humans with this SNP.^11^ This suggests that focusing on variants with low‐to‐rare frequencies could result in the identification of additional genes associated with complex traits.^12^ Similarly, common variants can contribute to rare congenital skeletal dysplasia, such as scoliosis or craniosynostosis, to account for the phenotypic variation that could not be fully explained by a rare pathogenic allele.^13^, ^14^

We hypothesized that genes contributing to biological pathways that regulate BMD could be identified by studying subjects at the phenotypic extremes for BMD. Our experimental approach to investigate this hypothesis utilized ES to determine the contribution of rare variants to low‐ or high‐BMD phenotypes. We used a gene‐based association test to evaluate the aggregated burden of these variants that potentially influence gene function in BMD physiology. Particular gene sets, such as the group of disease genes contributing to osteogenesis imperfecta (OI), were examined for a significant enrichment of rare variants. We sought to: (i) facilitate a more unified map of the pathways regulating BMD in men and potentially the major genes contributing to altered BMD in the population studied; (ii) better define the genetic factors contributing to BMD in older men; and (iii) facilitate disease‐risk prediction, prevention, and therapy.

## Materials and Methods

### Sample selection

MrOS is a multicenter prospective, longitudinal, observational study of risk factors for fractures in older men^15^ (http://mrosdata.sfcc-cpmc.net). Consent for DNA collection and analysis was obtained at each of six participating clinical sites, and the ES study was approved by the institutional review boards at the Baylor College of Medicine, Kennedy Krieger Institute, University of Montreal, Oregon Health & Science University, and UTHealth School of Public Health. The MrOS study included ambulatory men aged 65 years and above who were able to provide self‐reported data, lived close to the clinical site, and did not have health conditions that could cause imminent death. Recruitment details can be found in a previous report.^16^ Exclusion criteria were inability to walk without the assistance of another person and the absence of at least one native hip available for BMD measures.^15^ From this MrOS cohort, we selected individuals who had two BMD measurements of the hip demonstrating <5% variability by either a DXA scan or a quantitative computed tomography (QCT). We then ranked these individuals by age and BMI‐adjusted total hip BMD. We selected those individual subjects with BMD values >3 SD above (*n* = 13) or below (*n* = 3), then further selected individuals with the highest 100 (representing 3% of the remaining distribution) and lowest 100 (representing 2.6% of the remaining distribution) BMDs.

All of the individuals included had undergone SNP genotyping, which indicated a European ancestry.

### Sequencing and variant filtering

ES was performed as described previously^17^, ^18^ on 110 individuals from the high‐BMD group and 98 individuals from the low‐BMD group. Eighty‐two control samples were identified from the US Utah residents with Northern and Western European ancestry (CEU) population of the 1000 Genomes Project. Genomic DNA was isolated from peripheral blood monocytes. Exomes were captured from the Nimblegen's SeqCap EZ HGSC VCRome library (Roche NimbleGen, Madison, WI, USA). Sequencing was performed with the Illumina HiSeq 2000 platform (Illumina, San Diego, CA, USA). ES data were aligned and processed per the MERCURY pipeline as described previously.^19^ We performed joint calling using PLATYPUS v 0.7.9.1,^20^ and variants were brought forward for further analyses if they passed the quality filters and were annotated to have a potential protein consequence (ie, variant classification as stop‐loss, stop‐gain, nonsynonymous, InDel, splicing) using ANNOVAR.^21^, ^22^ Given the relatively small size of the sample, we limited our analysis to 51 known Mendelian disease genes implicated in BMD (Supplementary Table S2) based on the Online Mendelian Inheritance in Man (OMIM) database and our own literature review, specifically genes associated with autosomal dominant (AD) OI, autosomal recessive (AR) OI, connective tissue disorders, and osteosclerosis. We examined the variants that were found at <1% minor allele frequency (MAF) in the Exome Aggregation Consortium (ExAC) database for association with extremely low or high BMD.^20^ We analyzed variants found in ClinVar (http://www.ncbi.nlm.nih.gov/clinvar/) for known disease correlations.^23^

### Statistical analysis

For rare variants, defined as <1% MAF in the ExAC database, the National Heart, Lung, and Blood Institute (NHLBI) GO Exome Sequencing Project (ESP), and the CEU sample of the 1000 Genomes Project, we examined variants that were found in more than one case in the low or high BMD group. We performed the Fisher's exact test to evaluate whether the presence of rare variants in any of the 51 genes was associated with low or high BMD phenotypes by comparing extreme BMD groups with controls, respectively. For burden analyses, we performed a sequence kernel association test (SKAT) analysis^24^ on the 51 genes, including any variants that passed a quality filter. We used BMD as a dichotomous trait (high versus low) with age as a covariate for the SKAT analysis. We corrected for multiple comparisons by using a Bonferroni correction for 51 genes. We examined 51 individual genes and then grouped them into three groups: (i) *COL1A1*/*COL1A2* for AD OI; (ii) *CRTAP*, *FKBP10*, *LEPRE1*, *PPIB*, *SERPINF1*, *P3H1*, *SERPINH1*, *SP7*, *TMEM38B*, *WNT1*, *BMP1*, *SPARC* for AR OI; and (iii) *COL5A1*/*COL5A2* for Ehlers‐Danlos syndrome (EDS) classic type. We compared, by applying the Fisher's exact test, the proportion of variants observed in the low‐BMD group with the proportion of variants observed in the controls. Bonferroni correction was applied to the multiple comparison of the three gene sets; a *p* value <0.0167 was considered as statistically significant.

## Results

Within our cohort of subjects, we first examined the demographic characteristics of our patient cohort. We observed that there were significant differences in BMD and fracture rate between individuals classified as having high versus low BMD (Table 1). This suggests that high BMD is associated with a protective effect on lifetime fracture incidence, consistent with previous observations.^4^

**Table 1:** Demographic Characteristics of the MrOS Participants Included in the Sequencing Analysis

		Low BMD	High BMD	Statistical significance
Descriptive data	Number, *n*	98	110	
	Age, years	74.8 ± 6.7	73.8 ± 5.7	NS§
	BMI, kg/m^2^	27.8 ± 4.5	27.6 ± 3.2	NS§
BMD	Proximal femur, g/cm^2^	0.696 ± 0.083	1.263 ± 0.095	<0.00001§
	Femoral neck, g/cm^2^	0.588 ± 0.073	1.046 ± 0.126	<0.00001§
	Lumbar spine, g/cm^2^	0.953 ± 0.187	1.537 ± 0.278	<0.00001§
History of clinical fragility fracture	Hip, *n* (%)	25 (26%)	1 (1%)	<0.001*
	Spine, *n* (%)	12 (12%)	0	<0.001*

Individuals are all male and of European ancestry (based on self‐reported and SNP genotyping data). The samples in the two groups (low BMD versus high BMD) represent a similar distribution for age.

§ denotes Student's *t* test for statistical analysis.

* denotes Fisher's exact test for statistical analysis.

MrOS = Osteoporotic Fractures in Men Study.

Of the rare variants in the 51 known Mendelian genes implicated in high or low BMD, we found one individual who had a previously described variant in *LRP5* (chr11:68131252G > A, c.724G > A, p.Ala242Thr). This variant was previously reported to cause osteosclerosis in four families.^23^ Our subject with this variant demonstrated the highest BMD in our cohort, corresponding to a *Z*‐score of 5.2, consistent with the clinical phenotypes in the report.^25^

To identify recurrent variants that are potentially linked to extreme bone phenotypes, we examined the burden of rare variants in the 51 genes. We selected variants that were seen more than once in either the low or high BMD group and performed a Fisher's exact test. The result suggests a nominal significant association between these single‐nucleotide variants (SNVs) and an extreme BMD phenotype (Supplementary Table S1).

We applied Fisher's exact test to search for genes in which the presence of rare variants present a nonrandom association with low or high BMD. No significant single‐gene association was found after adjusting for multiple testing for the 51 known BMD genes (Supplementary Table S2). We then performed burden analyses with SKAT to search for genes associated with extreme BMD values. *WNT1* displayed the lowest *p* value (SKAT *p* = 0.015, Supplementary Table S3), though not reaching Bonferroni adjusted *p* value. As reported before, defects of WNT1 cause diseases with a semidominant inheritance pattern.^26^, ^27^ Biallelic variants, as compound heterozygous or homozygous alleles, in *WNT1* can cause OI type XV,^26^, ^27^, ^28^ an OI AR disease trait, whereas heterozygous variants can cause early‐onset osteoporosis as an apparent AD disease trait.^27^, ^28^ We identified rare variants in *WNT1* associated with low BMD, implicating *WNT1* variants in osteoporotic pathophysiology.

To characterize whether particular pathways or gene sets are significantly associated with extreme BMD phenotypes, we examined specific gene groups: AD OI trait type genes, AR OI trait type genes, and connective tissue disorder EDS genes. The dominant OI type genes, including *COL1A1* and *COL1A2*, did not show a significant association. However, heterozygous rare variant carrier states for recessive OI (*CRTAP*, *FKBP10*, *LEPRE1*, *PPIB*, *SERPINF1*, *SERPINH1*, *SP7*, *TMEM38B*, *WNT1*) demonstrated an approximately threefold increased frequency in individuals with low BMD compared with controls (*p* = 0.009). Variants in genes that cause EDS or EDS‐like phenotypes such as *SLC39A13*, *B4GALT7*, *COL3A1*, *COL5A1*, and *COL5A2* showed a trend of approximately twofold increased frequency in cases compared with controls (*p* = 0.031) (Table 2).

**Table 2:** Enrichment of Rare Variants in Recessive OI and EDS Gene Sets for the Low BMD Group

Gene set	Low BMD subjects with rare variants (MrOS)	Controls with rare variants (CEU)	*p* value
*COL1A1*/ *COL1A2*	2 (2.22%)	2 (2.44%)	1
AR OI Genes	16 (17.78%)	4 (4.88%)	0.009
EDS Genes	19 (21.11%)	7 (8.54%)	0.031

Number of samples with rare variants in genes belonging to a particular gene set displayed. *p* value for the three groups was calculated with Fisher's exact test. Total number of low BMD subjects is 98, and total number of controls is 82.

OI = osteogenesis imperfecta; EDS = Ehlers‐Danlos syndrome; MrOS = Osteoporotic Fractures in Men Study; CEU = US Utah residents with Northern and Western European ancestry; AR = autosomal recessive.

## Discussion

Our study aimed to identify rare variants that are associated with extremely low or high BMD in men over 65 years of age. Taking advantage of ES, we were able to validate previously reported pathogenic variants and identify rare genetic variants (SNV and Indels) and potential gene sets that are associated with extreme BMD phenotypes.

We focused on rare variants in our selected groups because of the rarity of such severe bone mass changes (>3 SD for BMD) in the population. As previously noted, the odds ratio or effect size is inversely associated with the population frequency of the variant. The observation of a pathogenic *LRP5* variant in our high‐BMD subject suggested that extreme phenotypes could be caused by variants with effect sizes close to those of Mendelian causal variants. Identification of these variants can serve as an efficient way for identifying genes implicated in the pathophysiological processes of producing such complex traits or diseases.

Analysis of individual variants or genes for genome‐wide association had the drawback that it overlooks the contribution of the network of genes that are orchestrated to perform interdependent functions in determining phenotypic variation or disease susceptibility. To understand the contribution of gene pathways,^29^ we examined gene sets for mutation burden in relation to AD OI, AR OI, and classic type EDS disease traits.^29^ We found a significant enrichment of rare variants in AR OI genes for low BMD, suggesting pathogenesis of these variants in affecting metabolic regulation of bone mass. This underlies the importance of Mendelian disease genes in contributing to a common disease trait, probably through a polygenic and multifactorial mechanism^30^, ^31^ in the current MrOS patient cohort. Of note, heterozygous variants for a biallelic locus tied to AR OI, *WNT1*, have been shown to contribute to the complex BMD quantitative trait.^27^, ^28^ Interestingly, these AR OI disease trait genes encode for collagen modification enzymes that are associated with collagen hydroxylation, glycosylation, and ultimately, crosslinking. Recent preclinical studies not only show these genes important in determining bone mass via alteration of TGF‐β signaling,^32^ but also potentially bone quality via crosslinking defects.^33^, ^34^, ^35^

Also of note, the EDS gene set showed significant associations with low BMD, which is compatible with the clinical observation that cohorts of EDS patients displayed significantly increased prevalence of vertebral fractures.^36^, ^37^, ^38^ Of the EDS genes we examined, *SLC39A13* and *B4GALT7* are known to cause spondylodysplastic EDS, where short stature and pathognomonic radiological findings were included as the diagnostic criteria.^39^ The inclusion of skeletal findings for the EDS diagnosis suggests convergence of the biological pathways regulating skeletal, tendon, ligament, and skin development. Interestingly, several groups have reported EDS‐like phenotypes caused by classical OI genes such as *COL1A1*.^40^, ^41^ Variants contributing to the EDS‐like phenotypes in atypical OI patients encompass a mosaic in‐frame deletion, frame‐shift deletion, or missense mutation in *COL1A1*. It is suspected that these mutations generate a hypomorphic protein, as opposed to the dominant negative form identified in dominant OI diseases. This is not surprising as there are significant overlapping clinical features between the phenotypes of OI and EDS patients in the context of heritable disorders of connective tissue.

Prediction scores for BMD have been developed based on significant SNPs from meta‐analyses of large GWASs on BMD.^42^ GRS63, one of the scores including 63 GWAS signals^42^ was associated with BMD (approximately 3% variation explained) but not BMD changes.^43^ The association between GRS63 and fractures was confirmed; however, this association was substantially reduced after adjusting for the effect of BMD.^43^ Our findings of a significant association between AR OI and EDS genes with low BMD suggest the possibility of developing a BMD prediction model based on aggregated rare mutation burden. However, the utility of such a model in predicting fractures needs to be carefully examined as the BMD‐adjusted GRS63 effect is limited for fracture prediction.^43^ In addition to GWASs, public genetic databases such as ExAC and the Genome Aggregation Database (gnomAD) are readily available for variant filtering (as we did here using 1% MAF as a cutoff), prioritizing (as we did with Fisher's exact test, see Supplementary Table S1), and interpreting. However, such an evaluation should be done very carefully, recognizing that the differences in genetic backgrounds and sequencing technique specifications between case studies (our cohort) and the control group (ExAC cohort) could severely interfere in the interpretation of observed statistics.^44^ For future investigations with higher statistical power and better clinical interpretation, not only the number of case studies needs to be increased, but also the control group has to be refined to match the settings of case studies.

Our pilot study implicates rare variant alleles in known Mendelian disease traits (OI and EDS) are associated with extreme low BMD in men over 65 years of age. We also provide insight into possible gene sets important for bone mass preservation such as recessive OI genes or classic type EDS genes. Utilizing this information may enable gene panel‐based screening as a biomarker for identifying the at‐risk male population for low BMD or osteoporosis.

## Disclosures

JRL has stock ownership in 23andMe, is a paid consultant for Regeneron Pharmaceuticals and Novartis, is a member of the Scientific Advisory Board of Baylor Genetics (BG), and is a co‐inventor on multiple United States and European patents related to molecular diagnostics for inherited neuropathies, eye diseases, and bacterial genomic fingerprinting. The Department of Molecular and Human Genetics at Baylor College of Medicine derives revenue from the chromosomal microarray analysis (CMA) and clinical exome sequencing offered at the Baylor Genetics website (BG: https://www.baylorgenetics.com/).

All other authors have no conflicts of interest to declare associated with the work presented in this manuscript.

## Authors' roles

SC, MJ, SJ, ZCA, PC, PFK, CMN, DMM, EB, RAG, ESO, JRL, JEP, and BL participated in the study concept and experimental design. SC, MJ, SJ, ZCA, PC, RFK, CNN, and DMM performed experiments and interpreted the data. SC and MJ wrote the first draft of the manuscript. All authors read and approved the final manuscript.

## Supporting information


**Supplemental Table S1.** Recurrent variants in low BMD or high BMD groups. This table shows recurrent rare variants identified in low BMD or high BMD group. *Pp*‐value is produced by applying the Fisher's exact test when comparing to the ExAC database.
**Supplemental Table S2.** Genes implicated in low or high bone density were examined for the burden of rare variants. Fifty‐one51 genes known for being associated with low or high BMD were examined for whether any harbors higher mutation burden for rare variants than expected using Fisher'’s exact test. The burden tests were performed on low BMD samples or high BMD samples compared to controls separately. Statistical test results for three AR OI and low BMD genes: *P3H1*, *SPARC* and *BMP1* were not shown as there were no rare variants found in these genes in either low or high BMD group or controls.
**Supplemental Table S3.** *WNT1* shows nominal significance for association with low BMD. We found a nominal association of rare variants in *WNT1* in the low BMD group (SKAT pp = 0.0149). The table represents these rare variants in *WNT1* in the low BMD group.
