# Supplementary material for: Genetic Burden Contributing to Extremely Low or High Bone Mineral Density in a Senior Male Population From the Osteoporotic Fractures in Men Study (MrOS)
Source: JBMR Plus. 2020 Jan 22;4(3):e10335. doi: 10.1002/jbm4.10335 (PMC7059823; doi:10.1002/jbm4.10335)
Supplement: Supplementary file 1 — Supplemental Table S1. Recurrent variants in low BMD or high BMD groups. This table shows recurrent rare variants identified in low BMD or high BMD group. Pp‐value is produced by applying the Fisher's exact test when comparing to the ExAC database. Supplemental Table S2. Genes implicated in low or high bone density were examined for the burden of rare variants. Fifty‐one51 genes known for being associated with low or high BMD were examined for whether any harbors higher mutation burden for rare variants than expected using Fisher'’s exact test. The burden tests were performed on low BMD samples or high BMD samples compared to controls separately. Statistical test results for three AR OI and low BMD genes: P3H1, SPARC and BMP1 were not shown as there were no rare variants found in these genes in either low or high BMD group or controls. Supplemental Table S3. WNT1 shows nominal significance for association with low BMD. We found a nominal association of rare variants in WNT1 in the low BMD group (SKAT pp = 0.0149). The table represents these rare variants in WNT1 in the low BMD group. [file JBM4-4-e10335-s001.docx]

**Supplemental Data**

**Supplemental Table 1. Recurrent variants in low BMD or high BMD groups.** This table shows recurrent rare variants identified in low BMD or high BMD group. P-value is produced by applying the Fisher's exact test when comparing to the ExAC database.

| Gene | Phenotype | Chr | Position | Minor  Allele | Major  Allele | Protein Effect | PolyPhen prediction | Case: Minor Allele # | Case: Major Allele # | Control: Minor Allele # | Control: Major Allele # | P value |
| --- | --- | --- | --- | --- | --- | --- | --- | --- | --- | --- | --- | --- |
| *COL5A2* | High BMD | 2 | 189927592 | A | G | NP_000384.2:p.Pro659Leu | 0.986 | 2 | 218 | 58 | 121208 | 0.004332 |
| *SERPINF1* | Low BMD | 17 | 1673303 | G | C | NP_002606.3:p.Ser81Tyr | 1 | 2 | 194 | 115 | 121294 | 0.01576 |
| *LRP5* | Low BMD | 11 | 68192736 | T | C | NP_002326.2:p.Arg1135Cys | 1 | 2 | 194 | 32 | 116534 | 0.01576 |
| *SERPINH1* | Low BMD | 11 | 75277974 | A | C | NP_001193943.1:p.Arg194Ser | 0.018 | 2 | 194 | 72 | 116406 | 0.01578 |
| *WNT1* | Low BMD | 12 | 49373410 | A | T | NP_005421.1:p.Ser88Arg | 0.174 | 4 | 192 | 353 | 119352 | 0.01862 |
| *TNFSF11* | Low BMD | 13 | 43148519 | G | A | NP_003692.1:p.Glu27Gly | 0.455 | 2 | 194 | 169 | 12990 | 0.0372 |
| *LEPRE1* | High BMD | 1 | 43220563 | C | T | NP_071751.3:p.Asp441Gly | 0.989 | 3 | 217 | 311 | 121390 | 0.04796 |

**Supplemental Table 2. Genes implicated in low or high bone density were examined for the burden of rare variants.** 51 genes known for being associated with low or high BMD were examined for whether any harbors higher mutation burden for rare variants than expected using Fisher’s exact test. The burden tests were performed on low BMD samples or high BMD samples compared to controls separately. Statistical test results for three AR OI and low BMD genes: *P3H1*, *SPARC* and *BMP1* were not shown as there were no rare variants found in these genes in either low or high BMD group or controls.

|  | | Low BMD Group vs. Controls | | High BMD Group vs. Controls | |  |
| --- | --- | --- | --- | --- | --- | --- |
| Gene | Impact on BMD | Fisher’s exact P-value | #Cases with vs. without rare variants | Fisher’s exact P-value | #Cases with vs. without rare variants | #Controls with variants : #Total |
| *ALPL* | low BMD | >0.999 | 0:90 | >0.999 | 0:99 | 0:82 |
| *B4GALT7* | low BMD | >0.999 | 2:88 | >0.999 | 0:99 | 1:81 |
| *COL1A1* | low BMD | >0.999 | 2:88 | >0.999 | 3:96 | 2:80 |
| *COL1A2* | low BMD | 0.05 | 0:90 | >0.999 | 4:95 | 4:78 |
| *COL3A1* | low BMD | >0.999 | 3:87 | >0.999 | 3:96 | 2:80 |
| *COL5A1* | low BMD | 0.172 | 7:83 | 0.115 | 8:91 | 2:80 |
| *COL5A2* | low BMD | >0.999 | 3:87 | 0.296 | 6:93 | 2:80 |
| *CREB3L1* | low BMD | >0.999 | 0:90 | >0.999 | 0:99 | 0:82 |
| *CRTAP* | low BMD | >0.999 | 2:88 | >0.999 | 1:98 | 1:81 |
| *FBN1* | low BMD | 0.749 | 6:84 | >0.999 | 4:95 | 4:78 |
| *FGFR3* | low BMD | 0.498 | 2:88 | >0.999 | 1:98 | 0:82 |
| *FKBP10* | low BMD | 0.498 | 2:88 | 0.252 | 3:96 | 0:82 |
| *IFITM5* | low BMD | >0.999 | 0:90 | >0.999 | 0:99 | 0:82 |
| *LEPRE1* | low BMD | >0.999 | 2:88 | 0.628 | 3:96 | 1:81 |
| *LRP5* | low BMD | 0.722 | 5:85 | 0.73 | 5:94 | 3:79 |
| *PLOD2* | low BMD | >0.999 | 1:89 | >0.999 | 1:98 | 1:81 |
| *PLOD3* | low BMD | >0.999 | 4:86 | 0.33 | 1:98 | 3:79 |
| *PLS3* | low BMD | >0.999 | 0:90 | >0.999 | 1:98 | 0:82 |
| *PPIB* | low BMD | 0.226 | 0:90 | 0.204 | 0:99 | 2:80 |
| *SERPINF1* | low BMD | 0.247 | 3:87 | 0.502 | 2:97 | 0:82 |
| *SERPINH1* | low BMD | 0.073 | 4:86 | 0.502 | 2:97 | 0:82 |
| *SLC34A1* | low BMD | 0.214 | 5:85 | >0.999 | 2:97 | 1:81 |
| *SLC39A13* | low BMD | 0.073 | 4:86 | 0.628 | 3:96 | 0:82 |
| *SLC9A3R1* | low BMD | >0.999 | 0:90 | >0.999 | 1:98 | 1:81 |
| *SP7* | low BMD | 0.498 | 2:88 | >0.999 | 1:98 | 0:82 |
| *TMEM38B* | low BMD | 0.498 | 2:88 | 0.502 | 2:97 | 0:82 |
| *TNFRSF11B* | low BMD | >0.999 | 0:90 | >0.999 | 1:98 | 0:82 |
| *TAPT1* | low BMD | >0.999 | 0:90 | 0.502 | 2:97 | 0:82 |
| *TRPV4* | low BMD | >0.999 | 3:87 | >0.999 | 4:95 | 3:79 |
| *WNT1* | low BMD | 0.247 | 3:87 | >0.999 | 0:99 | 0:82 |
| *SNX10* | high BMD | >0.999 | 0:90 | >0.999 | 0:99 | 0:82 |
| *SLC29A3* | high BMD | 0.226 | 0:90 | 0.204 | 0:99 | 2:80 |
| *IKBKG* | high BMD | >0.999 | 0:90 | 0.502 | 2:97 | 0:82 |
| *KIND3* | high BMD | 0.226 | 0:90 | 0.204 | 0:99 | 2:80 |
| *ANKH* | high BMD | >0.999 | 2:88 | >0.999 | 1:98 | 1:81 |
| *CA2* | high BMD | >0.999 | 1:89 | >0.999 | 1:98 | 0:82 |
| *CLCN7* | high BMD | >0.999 | 1:89 | >0.999 | 1:98 | 0:82 |
| *CTSK* | high BMD | >0.999 | 0:90 | >0.999 | 1:98 | 0:82 |
| *FAM123B* | high BMD | >0.999 | 0:90 | >0.999 | 1:98 | 1:81 |
| *FAM20C* | high BMD | >0.999 | 0:90 | >0.999 | 1:98 | 0:82 |
| *LEMD3* | high BMD | >0.999 | 1:89 | >0.999 | 1:98 | 1:81 |
| *OSTM1* | high BMD | >0.999 | 0:90 | >0.999 | 0:99 | 0:82 |
| *SOST* | high BMD | >0.999 | 0:90 | >0.999 | 0:99 | 0:82 |
| *TCIRG1* | high BMD | 0.738 | 4:86 | 0.471 | 3:96 | 5:77 |
| *TGFB1* | high BMD | >0.999 | 0:90 | >0.999 | 2:97 | 1:81 |
| *TNFRSF11A* | high BMD | >0.999 | 3:87 | >0.999 | 3:96 | 3:79 |
| *TNFSF11* | high BMD | >0.999 | 2:88 | >0.999 | 2:97 | 1:81 |
| *TYROBP* | high BMD | 0.477 | 0:90 | 0.453 | 0:99 | 1:81 |

**Supplemental Table 3. *WNT1* shows nominal significance for association with low BMD.** We found a nominal association of rare variants in *WNT1* in the low BMD group (SKAT p=0.0149). The table represents these rare variants in *WNT1* in the low BMD group.

| Gene | Phenotype | Chr | Position | Variant | Case: Minor Allele # | Case: Major Allele # | Control: Minor Allele # | Control: Major Allele # |
| --- | --- | --- | --- | --- | --- | --- | --- | --- |
| *WNT1* | Low BMD | 12 | 49373373 | c.227C>G, p.Pro76Arg | 1 | 195 | 1 | 120172 |
| *WNT1* | Low BMD | 12 | 49374230 | c.382T>C, p.Phe128Leu | 1 | 195 | Not observed | ~120000 |
| *WNT1* | Low BMD | 12 | 49375064 | c.754G>C, p.Gly252Arg | 1 | 195 | 43 | 64904 |
